# Comparative genomics and functional study of lipid metabolic genes in *Caenorhabditis elegans*

**DOI:** 10.1186/1471-2164-14-164

**Published:** 2013-03-12

**Authors:** Yuru Zhang, Xiaoju Zou, Yihong Ding, Haizhen Wang, Xiaoyun Wu, Bin Liang

**Affiliations:** 1 Key Laboratory of Animal Models and Human Disease Mechanisms, Kunming Institute of Zoology, Chinese Academy of Sciences, 32 Jiao-Chang Dong Road, Kunming, Yunnan 650223, China; 2 Department of Life Science and Biotechnology, Kunming University, Kunming 650214, China

**Keywords:** *Caenorhabditis elegans*, Lipid metabolism, Comparative genomics, RNAi, Fat storage

## Abstract

**Background:**

Animal models are indispensable to understand the lipid metabolism and lipid metabolic diseases. Over the last decade, the nematode *Caenorhabditis elegans* has become a popular animal model for exploring the regulation of lipid metabolism, obesity, and obese-related diseases. However, the genomic and functional conservation of lipid metabolism from *C*. *elegans* to humans remains unknown. In the present study, we systematically analyzed genes involved in lipid metabolism in the *C*. *elegans* genome using comparative genomics.

**Results:**

We built a database containing 471 lipid genes from the *C*. *elegans* genome, and then assigned most of lipid genes into 16 different lipid metabolic pathways that were integrated into a network. Over 70% of *C*. *elegans* lipid genes have human orthologs, with 237 of 471 *C*. *elegans* lipid genes being conserved in humans, mice, rats, and *Drosophila*, of which 71 genes are specifically related to human metabolic diseases. Moreover, RNA-mediated interference (RNAi) was used to disrupt the expression of 356 of 471 lipid genes with available RNAi clones. We found that 21 genes strongly affect fat storage, development, reproduction, and other visible phenotypes, 6 of which have not previously been implicated in the regulation of fat metabolism and other phenotypes.

**Conclusions:**

This study provides the first systematic genomic insight into lipid metabolism in *C*. *elegans*, supporting the use of *C*. *elegans* as an increasingly prominent model in the study of metabolic diseases.

## Background

Lipids are hydrophobic molecules with thousands of species including fatty acids and fatty acids derivates, e.g., triglycerides, phospholipids, sphingolipids, sterols, and the like. Generally, lipids function as energy storage, components of biological membrane, and signaling molecules, and likewise play a variety of biological roles in almost all aspects of life: growth, development, reproduction, stresses resistance, aging and longevity, etc. [1]. Accordingly, the homeostasis of lipid metabolism must be tightly regulated. Misregulation of lipid metabolism is often associated with many human diseases, particularly metabolic diseases, such as obesity, type 2 diabetes (T2D), cardiovascular diseases (CVD), and non-alcoholic fatty liver (NAFL) [1,2], all of which are increasingly prevalent both in both developed and developing countries, such as China [3].

Previous studies have illustrated that the human systems which control appetite, energy partitioning, and the integration of lipid metabolic processes are highly complex and redundant [4,5]. Therefore, employing animal models is indispensable in further understanding the lipid metabolism and lipid metabolic diseases. In some situations, lower and less complex organisms are particularly useful in providing simpler and clearer views of the fundamental regulation of lipid metabolism as opposed to higher, more complex organisms. Chiefly due to the advantages of rapid generation time over the last decade, easy genetic manipulation and visualization of lipid droplets at whole animal level has made the tiny nematode, *C*. *elegans* an emerging model for exploring the biological functions of lipids and the genetic basis of fat storage regulation. To date, research on this organism has uncovered numerous findings in lipids metabolism and the associated fundamental regulatory mechanisms [6-11], thereby prompting further exploration of its utility as an animal model in these types of studies.

As the first multicellular organism to have its genome completely sequenced [12] and further deeply analyzed [13]*C*. *elegans* possesses for biosynthesis of polyunsaturated fatty acids [14], mono-methyl branched fatty acids [15], ceramides [16], phospholipids [17-21], and also the encoding of many lipid binding proteins and transporters [22]. This organism, however, lacks some key genes involved in the biosynthesis of sterol, forcing the worm to obtain it from its diet [23,24]. To date, however, how many potential lipid metabolic genes and pathways do exist in the *C*. *elegans* genome is not known. Likewise and more importantly for its application as an animal model, the genomic and functional conservation of lipid metabolism from *C*. *elegans* to humans is unclear, limiting *C*. *elegans*’ utility for in depth exploration of the fundamental mechanisms of lipid metabolism and human metabolic diseases.

In the present study, we analyzed genes involved in lipid metabolism in the *C*. *elegans* genome by comparative genomics. We first constructed a database containing 471 lipid metabolic genes from the *C*. *elegans* genome, and further assigned most of these into different lipid metabolic pathways. Afterward, we systematically compared these genes in the human, *Drosophila*, mouse, and rat genomes. Ultimately, we took the advantage of RNA-mediated interference (RNAi) to disrupt the expression of most lipid metabolic genes, allowing us to explore the biological roles these play in *C*. *elegans*.

## Results

### Database construction of *C*. *elegans* lipid metabolic genes

**Figure 1:**
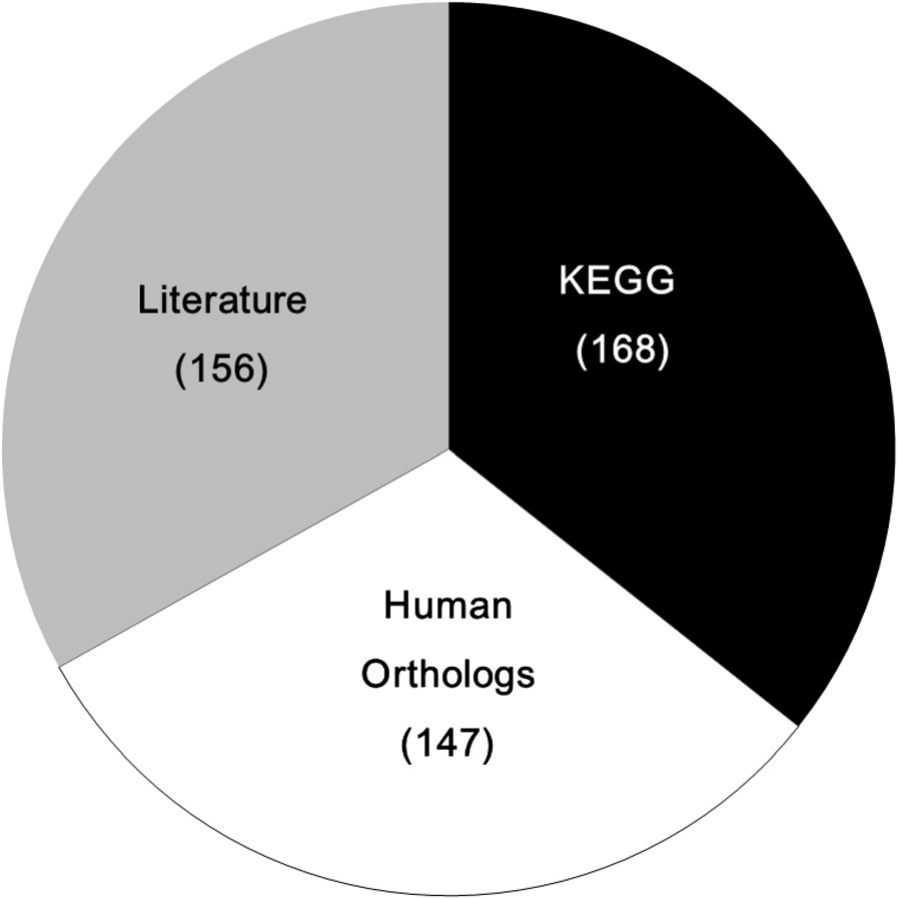
**Lipid metabolic genes in *C*. *elegans***. The *C*. *elegans* genome contains 471 lipid genes, including 168 genes taken from KEGG database colored black, 147 human orthologs colored white, and 156 genes manually picked from WormBase (http://www.wormbase.org) and the published literature colored gray.

To date, the KEGG database (http://www.genome.jp/kegg/) has collected 168 lipid metabolic genes from the *C*. *elegans* genome; this may be far from a complete listing, given the diversity and complexity of lipids in any organism. To garner a full list of lipid metabolic genes from the *C*. *elegans* genome, we retrieved lipid metabolic genes by manually searching the WormBase website (http://www.wormbase.org) and published papers collected from NCBI PubMed, as well as the *C*. *elegans* orthologs of 407 human lipid metabolic genes and 168 lipid genes from the KEGG database [22,25-28]. Eventually, 471 lipid metabolic genes were retrieved from the *C*. *elegans* genome including 147 orthologs of human lipid metabolic genes and 156 genes collected from the literature and WormBase (Figure 1 and Additional file 1: Table S1). In doing so, we constructed the first complete database of lipid metabolic genes of *C*. *elegans*, covering most potential lipid metabolic genes that could be found out in *C*. *elegans* genome at present (Figure 1 and Additional file 1: Table S1).

The biochemical function of the 471 lipid metabolic genes was further annotated with Ensembl BioMart (http://asia.ensembl.org/biomart/martview/dd89899865f812aff4dcd9ad0dfede49), though the 147 human orthologs and 168 lipid genes from KEGG were already annotated (http://www.genome.jp/kegg/). All 471 genes were also checked again in WormBase (Additional file 1: Table S1). Fuxrthermore, the function annotation obtained from ClueGo [29] showed that 471 lipid genes in *C*. *elegans* were not only involved in distinct lipid metabolic processes, but also play multiple roles in response to oxidative stress, aging and longevity, as well as other biological processes (Additional file 2: Figure S1).

Most of the 471 lipid genes in this database are predicted to encode specific enzymes that play roles in defined reactions, though experimental biochemical evidences for this conclusion may be lacking. Some genes predicted to encode the same or similar enzymes were classified into a family. However, they may actually have same, distinct, or no biological conversion/function, as well as may involve into one or more reactions (Table 1). For example, 7 fatty acid desaturase genes were present in the *C*. *elegans* genome, but they display completely different preference of substrates except *fat*-*6* and *fat*-*7*[14]. Cytochrome P450 enzymes are a superfamily of haem-containing mono-oxygenases with extraordinary diversity in their biochemical function across all kingdoms of life. Within the *C*. *elegans* genome, 81 full length cytochrome P450 (CYP) genes have been found, but the exact biological functions of the vast majority of CYPs are largely unknown as of yet [30]. Only 38 of the 81 CYPs may play a role in lipid metabolism, based on our findings from KEGG, WormBase, and the published literature. Another super family is lipase, consisting of 66 genes (Table 1), which may encode hormone-sensitive lipase, phospholipase A2, B1, C, D, triacylglycerol lipase, or unknown lipase (Additional file 1: Table S1).

**Table 1:** **Lipid metabolic gene families in *C*. *elegans***

**Gene**	**Enzyme**	**Number in database (all/mapped in pathways)**
*acdh*	acyl-CoA dehydrogenase	13 (13/13)
*ace*	acetylcholinesterase	4 (4/4)
*acl*	acyltransferase	14 (14/14)
*acox*	acyl-CoA oxidase	8 (8/8)
*acs*	acyl-CoA synthetase	22 (22/22)
*alh*	aldehyde dehydrogenase	13 (13/12)
*cpt*	carnitine palmitoyltransferase	6 (6/6)
*cyp*	cytochrome P450s	38 (81/35)
*dhs*	dehydrogenase	31 (31/9)
*dgat*	acyl-CoA:diacylglycerol acyltransferase	4 (4/4)
*ech*	enoyl-CoA hydratase	11 (11/11)
*elo*	elongase	9 (9/4)
*fat*	fatty acid desaturase	7 (7/7)
*lbp*	lipid binding protein	9 (9/9)
*lipl*/*lips*	lipase	66 (66/57)
*mboa*	lysophospholipid acyltransferase	7 (7/7)
*scrm*	scramblase	8 (8/0)
*stdh*	steroid dehydrogenase	4 (4/4)
*tat*	flippase	6 (6/0)
total		280 (323/226)

### 471 genes potentially involved in 16 lipid metabolic pathways in *C*. *elegans*

**Figure 2:**
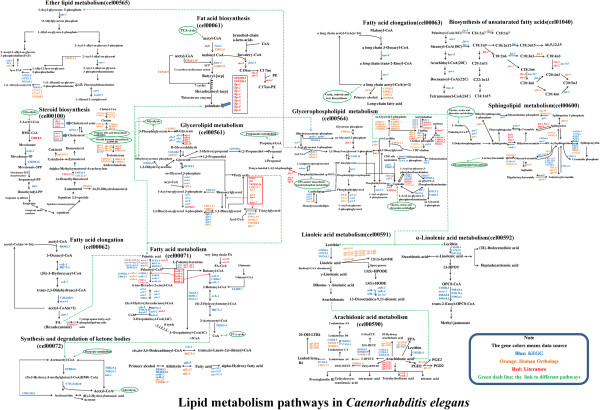
**Lipid metabolism pathways in *C*. *elegans*.** 16 lipid metabolism pathways are integrated into a lipid metabolism network. Genes were colored based on their data resources (see Figure 1). Black arrows linked different substrates. Two continuous black arrows indicate that there are more than two reactions. The dashed green arrow was used to link different pathways. The Green circle surround linked the pathway name.

Totally, 168 *C*. *elegans* lipid genes from the KEGG database were assigned into 15 lipid metabolic pathways: fatty acid biosynthesis [PATH:cel00061], fatty acid elongation in mitochondria [PATH:cel00062], fatty acid metabolism [PATH:cel00071], synthesis and degradation of ketone bodies [PATH:cel00072], steroid biosynthesis [PATH:cel00100], glycerolipid metabolism [PATH:cel00561], primary bile acid biosynthesis [PATH:cel00120], steroid hormone biosynthesis [PATH:cel00140], glycerophospholipid metabolism [PATH:cel00564], ether lipid metabolism [PATH:cel00565], sphingolipid metabolism [PATH:cel00600], arachidonic acid metabolism [PATH:cel00590], linoleic acid metabolism [PATH:cel00591], alpha-linolenic acid metabolism [PATH:cel00592], and biosynthesis of unsaturated fatty acids [PATH:cel01040]. To clearly distinguish the fatty acid elongation in the cytoplasmic compartment from that in the mitochondria compartment [PATH: cel00062], we renamed this pathway as fatty acid elongation [PATH: cel00063]. Collectively, we built 16 lipid metabolic pathways in *C*. *elegans* (Figure 2).

To gain a clearer picture of lipid metabolic pathways in *C*. *elegans*, all 16 pathways were further integrated into a lipid metabolism network (Figure 2). Additionally, 303 lipid genes including 147 human orthologs and 156 genes reported in the literature were assigned into the 16 pathways based on their annotated or reported biological function (Figure 2). However, due to a lack of both clear evidence of the precise biological function and detailed information, some genes could not be assigned into any pathway, even though their paralogs were assigned in the same pathway. Subsequently, we constructed the first lipid metabolism network with 16 pathways containing 471 lipid genes from *C*. *elegans* (Figure 2).

Only 35 of the 81 CYPs could be assigned into different lipid metabolic pathways (Figure 2). Based on KEGG annotation, *cyp*-*33*E2 may function in fatty acid metabolism, though it was reported to be involved in the biosynthesis of eicosanoides derived from eicosapentaenoic acid (EPA) [31]. Therefore, we reassigned *cyp*-*33*E2 into the arachidonic acid metabolism pathway (Figure 2). Despite *cyp*-*35*B1 being shown as a target of insulin/IGF-I-like signaling (IIS) [32], and *Cyp*-*31*A2 and *Cyp*-*31A3* being involved in the production of lipids required for eggshell formation [33], their real catalyzing reactions were unclear enough that they could not be assigned into any lipid metabolic pathway.

### Genomic comparison of lipid metabolic genes among humans, mice, rats, *Drosophila*, and *C*. *elegans*

Research on animal models has brought numerous powerful and valuable insights to both basic human biology and disease pathology [34-36]. However, under many circumstances, animal models fall short of our expectations, going so far as to result in contradictory or opposite findings. These discrepancies may be due in part to the changes in the conservation of genetically controlled pathways from human to model animals [36-38]. This reality spurred us to inquire about the conservation of lipid metabolism that exists between *C*. *elegans* and humans, as well as mice [39,40], rats [41,42], and *Drosophila*[43-46], all of which are commonly used as models to understand the mechanisms and potential therapies for human metabolic diseases.

All 471 *C*. *elegans* lipid genes were used to search for orthologs in the human, mouse, rat and *Drosophila* genomes using Ensembl BioMart (Methods). Eventually, 581, 585, 563, and 428 lipid genes respectively were obtained in the human, mouse, rat, and *Drosophila* genomes (Additional file 3: Table S2 and Additional file 4: Figure S2). Comparison of these lipid genes revealed that 237 genes are conserved in all five organisms. Over 70% of *C*. *elegans* lipid genes have orthologs in humans, mice, and rats (Table 2). Conversely, 72.12% (419/581) human lipid genes are conserved in *C*. *elegans* (Table 2), suggesting a high conservation of lipid metabolism genes between *C*. *elegans* and humans, as well as between *C*. *elegans* and both mice and rats.

**Table 2:** Conservation of lipid metabolic genes in 5 model organisms

**Organisms (lipid genes)**	***C*. *elegans *(471) Orthologs**	**Human (581) Orthologs**
*C*. *elegans* (471)	-	419(72.12%)
Human (581)	370(78.56%)	-
Mouse (585)	389(82.59%)	539(92.77%)
Rat (563)	375(79.62%)	517(88.98%)
*Drosophila* (428)	352(74.73%)	340(58.52%)

### Conservation of *C*. *elegans* lipid metabolic genes in human disease

**Figure 3:**
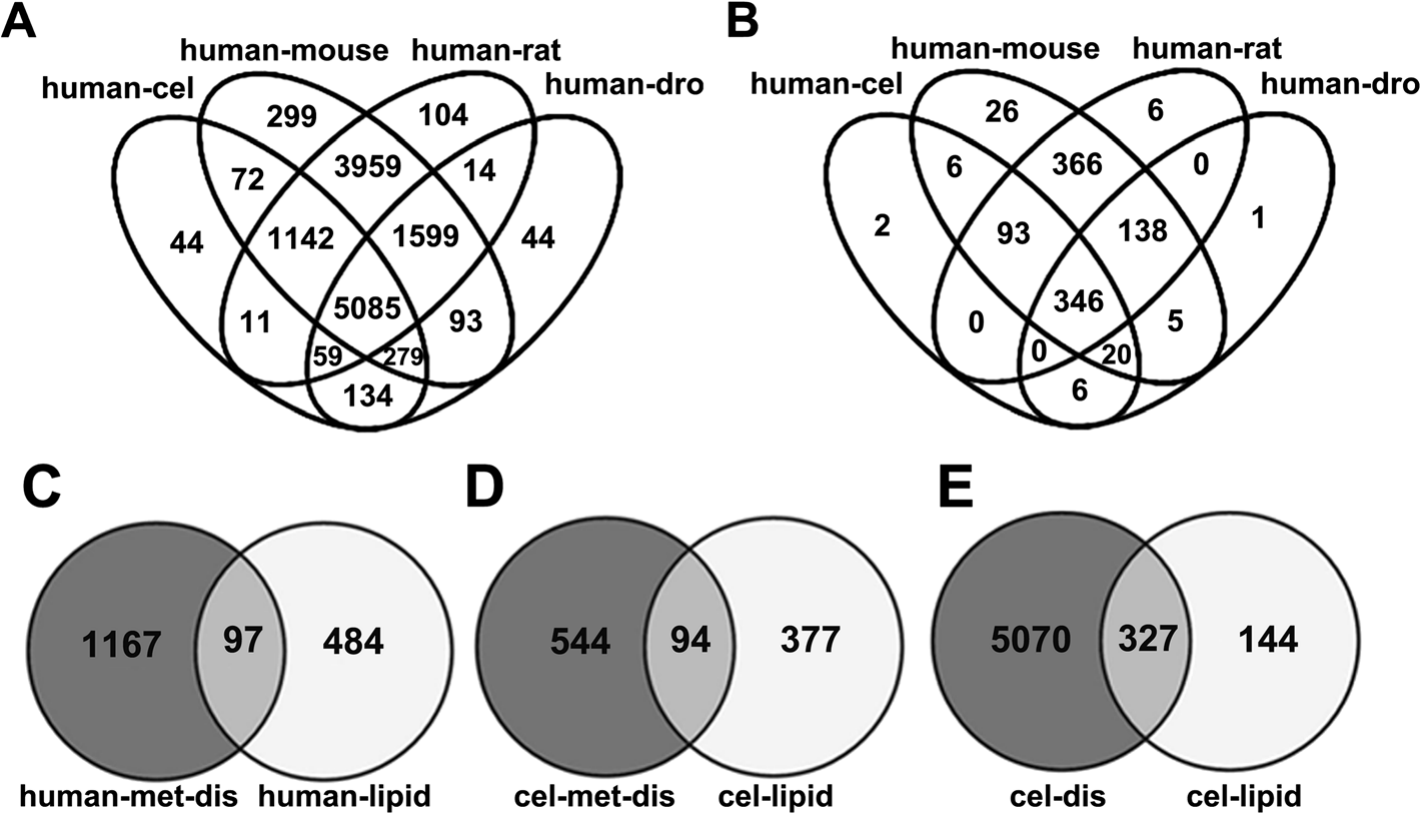
**Venn diagram of human disease genes orthologs and the relationship between lipid genes and metabolic disease associated genes. A**: Venn diagram of 15181 human disease gene orthologs made with VENNY. Numbers in the overlapping and non-overlapping areas of the diagram indicate the number of genes found by overlapping or unique sets of orthologs in each species; **B**: Venn diagram of 1264 human metabolic disease genes orthologs; **C**: Comparison of 581 human lipid genes with human metabolic disease genes (97 of 581 lipid genes overlapped with metabolic disease genes); **D**: Similarly to the phenomenon in human genes, 94 of 471 *C*. *elegans* lipid genes were related to metabolic disease; **E**: In *C*. *Elegans*, 327 lipid genes were associated with human diseases.

An imbalance of major lipid signaling pathways has previously been implicated in obesity, type 2 diabetes, atherosclerosis, hypertension, non-alcoholic fatty liver disease, etc. [1,47]. To analyze the relationship between lipid genes and human metabolic diseases, human disease genes were retrieved from the OMIM database (http://www.ncbi.nlm.nih.gov/omim/). At present, the OMIM database has collected 19,994 MIM IDs including genes, phenotypes, and locus which we downloaded from the newly updated NCBI FTP. Using Ensembl Biomart ID Mapping, we found 15,181 genes that could be considered human disease genes. Comparison between humans and the other four organisms revealed that mice, rats, *C*. *elegans*, and *Drosophila* have 13259, 12717, 5397, and 5889 respective orthologs, in which 5085 genes are conserved in all five organisms (Figure 3A). Furthermore, 1264 of these 15181 genes may be considered metabolic disease genes that were filtered within the database by keywords ‘Obesity, Diabetes, Metabolic disease, and others major obesity related diseases’. Similarly, 1152, 1051, 638, and 632 orthologs of human metabolic disease genes could be found among mouse, rat, *C*. *elegans*, and *Drosophila*, respectively, in which 346 orthologous genes are conserved in all five organisms (Figure 3B).

In humans, 97 of the 581 lipid genes are overlapped with metabolic disease genes (Figure 3C). Similarly, 94 of the 471 *C*. *elegans* lipid genes are orthologs of human metabolic disease genes (Figure 3D). Crucially, all 94 *C*. *elegans* lipid genes associated with metabolic diseases are completely overlapped with 97 human metabolic disease genes. In mice, rats, and *Drosophila*, respectively there are 128, 107, and 97 lipid genes associated with metabolic diseases, with 71 genes conserved across all five species (Additional file 5: Table S3). Moreover, 327 of the 471 lipid metabolism genes in *C*. *elegans* are also orthologs of human disease genes (Figure 3E), implying that great number of lipid genes are also involved in other disease processes aside from metabolic syndrome.

### Investigated of the biological functions of lipid metabolic genes in *C*. *elegans* by RNAi

Among the 471 C. *elegans* lipid metabolic genes, only a small number have been biochemically and functionally characterized, demonstration that they participate into growth, development, dauer formation, aging and longevity, stress responses, and the like. However, the biological functions of most lipid metabolic genes remain unknown. The current Argilent *C*. *elegans* RNAi library covers about 86% of the known and predicted genes, providing a powerful tool to further investigate the biological functions of the lipid genes. We opted to take advantage of RNA-mediated interference (RNAi) to disrupt the expression of each lipid gene and investigate easily visible phenotypes including growth, sterility, and fat storage, indicated by Nile Red staining of fixation [48].

**Figure 4:**
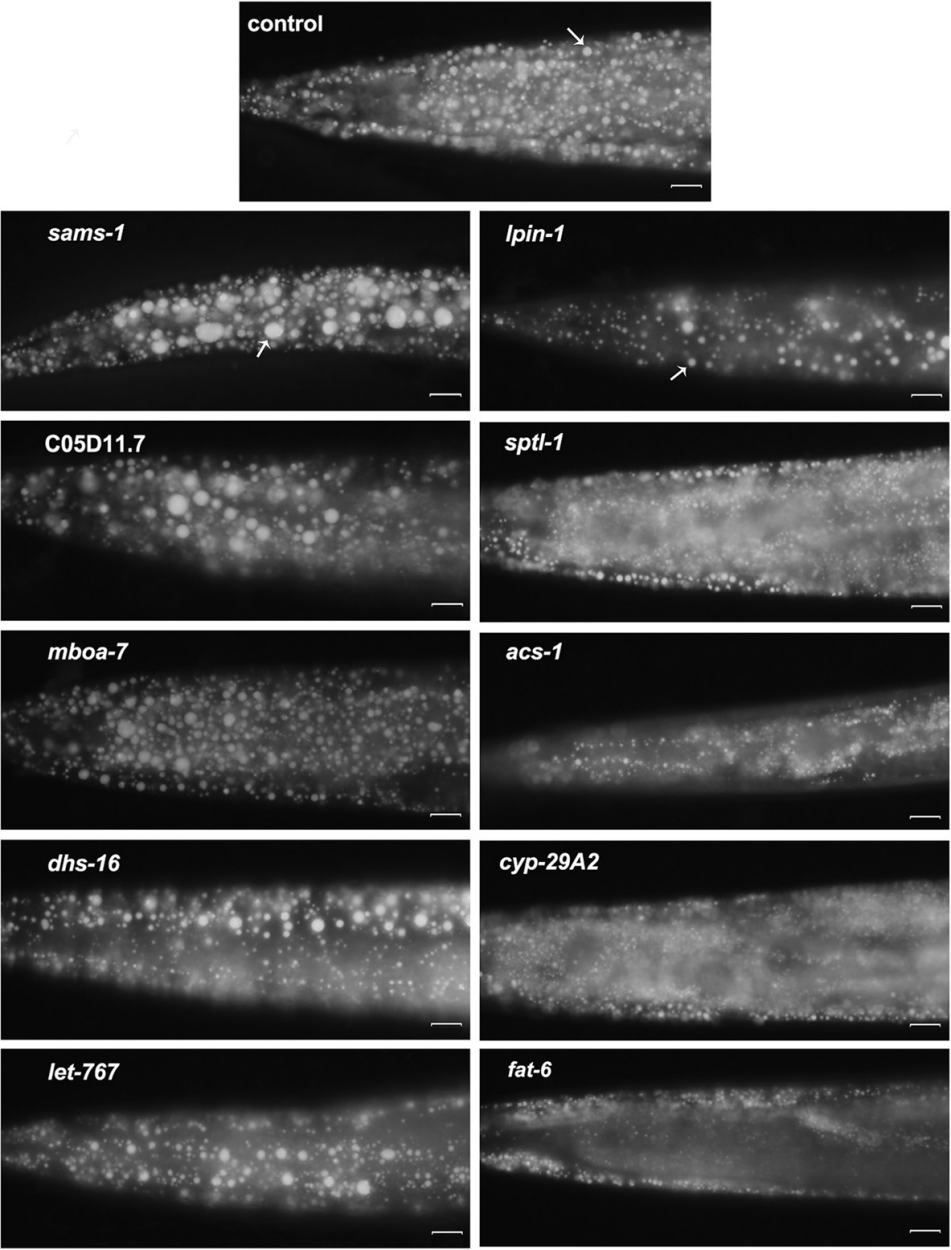
**Inactivation of 10 lipid genes by RNAi dramatically changed fat storage in *C*. *elegans*.** Late L4 worms were fixed with paraformaldehyde and stained with Nile Red [48]. Images were captured using identical settings and exposure time for each image. Inactivation of *sams*-*1*, *mboa*-*7*, and C05D11.7 led to increased lipid size; RNAi knockdown of *dsh*-*16* and *let*-*767* slightly though noticeably increased the size of lipid droplets in *C*. *elegans*; Meanwhile, RNAi inactivation of *lpin*-*1*, *sptl*-*1*, *acs*-*1*, *cyp*-*29*A2 and *fat*-*6* resulted in significantly decreased fat storage and comparatively smaller size of lipid droplets. Bar, 10 μm. Arrow indicates the lipid droplet.

Of the 471 lipid genes in *C*. *elegans*, 356 have available RNAi strains. Of those with available strains, RNAi knockdown of 21 genes consistently displayed remarkable phenotypes with significantly altered fat storage, growth defect, lethal, or sterility (Table 3), while10 of these 21 genes significantly affect fat storage. Inactivation of *sams*-*1*, *mboa*-*7*, and C05D11.7 led to large lipid droplet size (Figure 4). *sams*-*1* encodes S-adenosylmethionine synthetase (SAMS) involved in the biosynthesis of phosphocholine [PATH:Cel00564]. Mutation of *sams*-*1* displayed large size of lipid droplets [20,21]. *mboa*-*7* encodes a member of the membrane-bound O-acyltransferase family, and is required for incorporation of PUFAs into phosphatidylinositol (PI) [PATH:Cel00564] [18]. C05D11.7 is an ortholog of the human patatin-like phospholipase (PNPLA) gene family, catalyzing the hydrolysis of triglycerides in adipose tissue. LET-767 has been reported to be a major 3-ketoacyl-CoA reductase required for the production of monomethyl branched and long chain fatty acids [49]. DHS-16 is a novel 3-hydroxysteroid dehydrogenase regulating the production of dafachronic acids (DAs) [50]. In *C*. *elegans*, RNAi knockdown of *dsh*-*16* and *let*-*767* slightly but obviously increased the size of lipid droplets (Figure 4).

**Table 3:** Phenotypes of lipid metabolic genes inactivated by RNAi

**Sequence name**	**Gene name**	**LDs LD size**	**Other phenotypes**	**Pathway**	**Key references**
C49F5.1	*sams*-*1*	increased	Growth rate variant, lean, sterile	Glycerophospholipid metabolism	[20,21]
C05D11.7	*atgl*	increased		Glycerolipid metabolism	[51,52]
F14F3.3	*mboa*-*7*	increased		Glycerophospholipid metabolism	[17,18]
C10F3.2	*dhs*-*16*	increased	Egg laying defect, growth rate variant, lethal, slim	Primary bile acid biosynthesis	[50]
C56G2.6	*let*-*767*	increased	Growth rate variant, slim, lethal, egg laying defect	Steroid hormone biosynthesis,Linoleic acid metabolism,Biosynthesis of unsaturated fatty acids	[49,53] (4)
H37A05.1	*lpin*-*1*	decreased	Growth rate variant, slim, egg laying defect	Glycerophospholipid metabolism	[54,55]
C23H3.4	*sptl*-*1*	decreased	Egg laying defect, lethal,pale	Sphingolipid metabolism	[16]
F46E10.1	*acs*-*1*	decreased	Growth rate variant, slim, larvae arrest, lethal	Fatty acid metabolism, Glycerophospholipid metabolism	[56,57]
T19B10.1	*cyp*-*29*A2	decreased	Egg laying defect, lethal	Arachidonic acid metabolism	
VZK822L.1	*fat*-*6*	decreased	Growth rate variant, sterile, pale, slim	Biosynthesis of unsaturated fatty acids	[14,58-60]
T27F6.6			Egg laying defect	Sphingolipid metabolism	
ZC416.8	*cha*-*1*		Egg laying defect	Glycerophospholipid metabolism	[61-64]
K12H4.5			Reduced brood size, growth rate variant (l3-l4,72 h normal)	Glycerophospholipid metabolism	
F25B4.6	*hmgs*-*1*		Growth rate variant, larval arrest, larval lethal,sterile	Synthesis and degradation of ketone bodies	
F56D1.5	*dhs*-*5*		Egg laying defect	Steroid hormone biosynthesis	
T02G5.8	*kat*-*1*		Growth rate variant (L3,72 h normal)	Synthesis and degradation of ketone bodies	[65,66]
F32H2.5	*fasn*-*1*		Larval arrest, lethal	Fatty acid synthesis	[8,22,67]
W09B6.1	*pod*-*2*		Larval arrest, lethal	Fatty acid synthesis	[22,68,69]
F11E6.5	*elo*-*2*		Pale, slim	Fatty acid synthesis	[14,70]
D2024.3	*elo*-*3*		Pale, slim, egg laying defect	unclear	
T01E8.3	*plc*-*3*		Sterile	Glycerophospholipid metabolism	[71-73]

**Figure 5:**
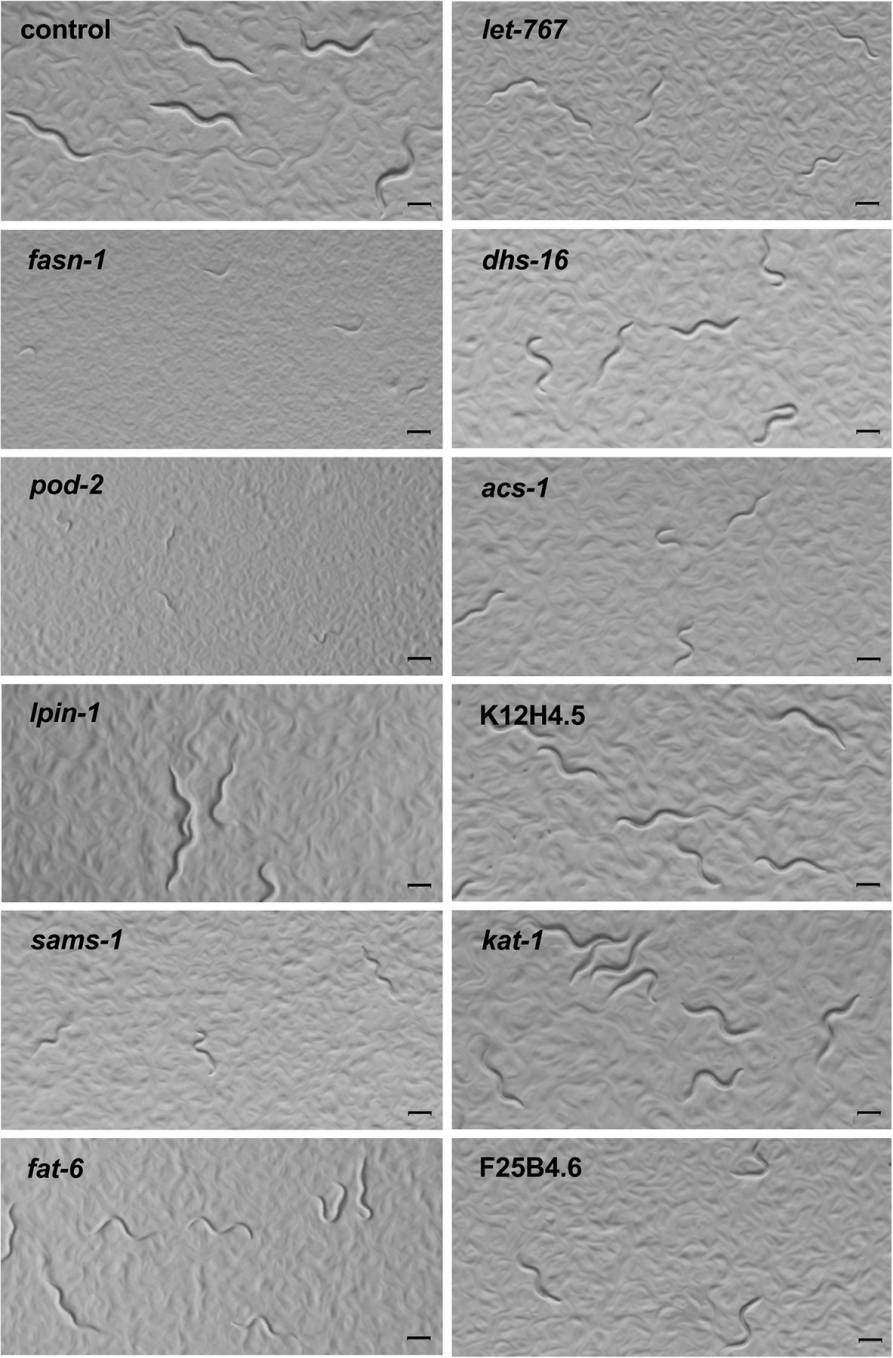
**Inactivation of 11 lipid genes by RNAi in *C*. *elegans *displayed growth and developmental defects.** RNAi of *fasn*-*1* and *pod*-*2* leads to severe larvae arrest. Inactivation of *lpin*-*1*, *sams*-*1*, *fat*-*6*, *let*-*767*, *dhs*-*16*, *acs*-*1*, K12H4.5, F25B4.6 and *kat*-*1* resulted in slower growth. All images were captured using identical settings and exposure time. Worms grown on *E*. *coli* HT115 containing an empty vector were used as control. Bar, 200 μm.

RNAi inactivation of *lpin*-*1*, *sptl*-*1*, *acs*-*1*, *cyp*-*29*A2 and *fat*-*6*, meanwhile, resulted in significantly decreased fat storage and a smaller size of lipid droplets. Additionally, inactivation of 9 genes led to slower growth (Figure 5). *pod*-*2* and *fasn*-*1* encode acetyl-CoA carboxylase and fatty acid synthase, respectively, and are predicted to catalyze the first and second step in de novo fatty acid biosynthesis [PATH:Cel00061]. Disruption of either of these two key genes by RNAi definitely led to both lethality and larval arrest (Figure 5). Meanwhile, inactivation of 13 other genes resulted in an egg laying variant (Table 3).

## Discussion

Understanding the lipid metabolism pathways and the involved genes is critical to further study of the biological functions of lipids and the mechanisms of metabolic diseases. A well-known animal model with a sequenced genome, like *C*. *elegans*, can provide a systemic genomic review for the whole lipid metabolism network. Several research groups have bioinformatically or functionally characterized one or several lipid metabolism pathways in *C*. *elegans*[14,16,22,26,68,74-76]. In our study, we created a database of 471 *C*. *elegans* lipid metabolism genes retrieved from the KEGG data, WormBase, and the published literature (Figure 1 and Additional file 1: Table S1). According to their annotation and real biological functions, these 471 genes could be designed into 16 lipid metabolism pathways integrated into a network (Figure 2). To our knowledge, ours is the first study to systematically analyze the lipid genes, metabolism pathways, and their biological functions in the animal model *C*. *elegans*.

Remarkably, aside from the 471 *C*. *elegans* lipid genes, we additionally found 581, 585, 563, and 428 respective lipid genes in the human, mouse, rat, and *Drosophila* genomes. Comparison of these genes revealed that 237 lipid genes are conserved in all five species. As such, this work provides useful information regarding lipid metabolism that will aid research utilizing *C*. *elegans* and other organisms as animal models. Among the 471 *C*. *elegans* lipid genes, 345 (73.25%) have orthologs in humans. Conversely, 380 of the 581 (65.4%) human lipid genes had orthologs in *C*. *elegans*. Notably, of 97 human lipid genes related to metabolic diseases, 94 have orthologs in *C*. *elegans* and 71 genes are conserved in the human, mouse, rate, *C*. *elegans* and *Drosophila* genomes, suggesting that lipid genes involved into metabolic diseases are highly conserved among model organisms. Altogether, these results indicate that *C*. *elegans* and humans share high conservation in terms of lipid genes and their roles in metabolic diseases, suggesting that *C*. *elegans* is likely a useful and insightful model for use not only in research on the fundamental biology of lipid metabolism, but also exploring the mechanisms of human metabolic diseases.

The *C*. *elegans* RNAi library provides numerous benefits to investigate the role of a specific gene. We thus systematically analyzed the biological functions of lipid genes with available RNAi strains. Among 471 lipid genes, nearly 40% genes were reported to affect phenotypes (http://www.wormbase.org), e.g. fat content, dauer, lifespan, brood size, growth, etc. In this study, we only focused on the clearly observable phenotypes of growth, sterility, and fat storage. However, due to the RNAi variation and gene redundancy of a family (Table 1), RNAi of many of the lipid genes displayed inconsistent, weak phenotypes that we excluded from this report. Consistently, however, inactivation of 21 lipid genes by RNAi remarkably affects worm fat metabolism, development, and other phenotypes. Of these 21 lipid genes, 6 genes T19B10.1 (*cyp*-*29A2*), T27F6.6, K12H4.5, F25B4.6 (*hmgs*-*1*), F56D1.5 (*dhs*-*5*) and D2024.3 (*elo*-*3*) have not been reported in literature (Table 3). Remarkbly, 19 out of 21 lipid genes (all except K12H4.5 and *dhs*-*5*; Table 3), are fairly conserved in humans and *C*. *elegans*. Moreover, C05D11.7, *fat*-*6*, *dhs*-*16*, *fasn*-*1*, *pod*-*2*, *elo*-*3*, and *lpin*-*1* have relationships with human metabolic diseases.

*C*. *elegans lpin*-*1* encodes a putative phosphatidic acid phosphatase orthologous to human LPIN1 (OMIM: 605518), LPIN2 (OMIM: 605519), and LPIN3 (OMIM: 605520) (http://www.wormbase.org). In humans and mice, Lpin-1 is associated with metabolic syndrome and type 2 diabetes [77-80] and mutations in human *LPIN2* cause Majeed syndrome [81]. *C*. *elegans lpin*-*1* has been implicated in affecting fat storage and the breakdown and assembly of the nuclear envelope [54,55]. Consistent with the mammalian *Lpin1* mutation, inactivation of *C*. *elegans lpin*-*1* by RNAi displayed resulted in low fat with small size of lipid droplets, as indicted by Nile Red staining (Figure 4). Human patatin-like phospholipase domain-containing 3 (PNPLA3) is associated with increased liver fat content and liver injury [82]. The *C*. *elegans* genome contains at least 5 orthologs of the patatin-like phospholipase (Additional file 1: Table S1). Interestingly, RNAi of C05D11.7, an ortholog of *PNPLA3* that was considered to encode adipose triglyceride lipase (ATGL) in *C*. *elegans*[51,52], led to higher fat and larger lipid droplets compared with the control in *C*. *elegans* (Figure 4), similar to other reports on humans and rodents [83,84].

Although *mboa*-*7* affects PUFA incorporation into PI [18], *dhs*-*16* regulates dafachronic acids metabolism [50], and *sptl*-*1* is involved in the biosynthesis of ceramide [16], they have not been previously implicated in fat storage. RNAi inactivation of *mboa*-*7* and *dhs*-*16* displayed increased lipid droplets size (Figure 4), whereas RNAi inactivation of *sptl*-*1*decreased lipid droplet size. The fat content of all three genes should likewise be measured by other methods (e.g., thin-layer chromatography and gas chromatography (TLC/GC)) in future studies. *acs*-*1* affects the incorporation of monomethyl branched chain fatty acid (C17iso) into phospholipids [56] and was reported to increase fat mass [57]. RNAi of *acs*-*1*, however, indeed decreased the density and size of lipid droplet (Figure 4). Inactivation of *let*-*767* by RNAi led to slower growth (Figure 5), but once the worms reached the late L4 or early young adult stage, they had increased lipid droplets size (Figure 4). Additionally, RNAi of *K12H4*.*5*, *kat*-*1*, and *F25B4*.*6* resulted in slower growth (Figure 5), but fat storage looked normal once the worm entered into late L4 or early young adulthood. Several members of cytochrome P450 superfamily were reported to functionally affect lipid metabolism [31,33,85,86]. We only found that RNAi of *cyp*-*29*A2 significantly decreased fat storage with small lipid droplets as compared with the control (Figure 4). Consequently, these lipid genes should potentially be considered as a starting point to further investigate their role in the regulation of fat storage.

## Conclusions

We created a database of 471 *C*. *elegans* lipid metabolism genes that could be designed into 16 lipid metabolism pathways integrated into a network. Additionally, 581, 585, 563, and 428 respective lipid genes in the human, mouse, rat, and *Drosophila* genomes were also retrieved. Over 70% of *C*. *elegans* lipid genes have human orthologs, with 237 of 471 *C*. *elegans* lipid genes being conserved in humans, mice, rats, and *Drosophila*, of which 71 genes are specifically related to human metabolic diseases. Furthermore, RNA-mediated interference (RNAi) of 21 lipid genes affects fat storage, development, and reproduction. This study provides the first genomic view on lipid metabolism in *C*. *elegans*, supporting the use of *C*. *elegans* as an increasingly prominent model in the study of metabolic diseases.

## Methods

### Retrieval of *C*. *elegans* lipid metabolic genes and database construction

Most of lipid metabolism genes used in this study were initially downloaded from the KEGG pathway database (http://www.genome.ad.jp/kegg/pathway.html), which has to date collected 168, 407, 383, 333, and 170 lipid metabolic genes respectively from the *C*. *elegans*, human, mouse, rat, and *Drosophila* genomes. Of the 407 human lipid genes present in the *C*. *elegans* genome, 364 were orthologs. Another 156 lipid metabolism genes in *C*. *elegans* were manually taken from WormBase (http://www.wormbase.org) and the published literature, mainly retrieved from PubMed (http://www.ncbi.nlm.nih.gov/pubmed/). Any overlapping genes were excluded with Perl program manually and online using the Venny program (http://bioinfogp.cnb.csic.es/tools/venny/ index.html). In total, 471 *C*. *elegans* lipid genes were been retrieved. The BioMart Interface (http://asia.ensembl.org/biomart/martview/53ac7015dbd78f9a5c2f0a3479fa4dc4) was applied to standardize all gene IDs.

### Identification of lipid metabolic genes in human, mouse, rat, and *Drosophila*

We used 471 *C*. *elegans* lipid metabolic genes to search for orthologs in the human, mouse, rat and *Drosophila* genomes by Ensembl BioMart and found 370, 389, 375, and 352 orthologs in human, mouse, rat, and *Drosophila*, respectively (Table 2). The KEGG database has collected 407 human lipid metabolic genes to date; after combining these and 370 human orthologs of *C*. *elegans* lipid genes, the overlapped genes were discarded. Within the human genome, 581 lipid genes have been found, and we used these in reversely to search for orthologs in *C*. *elegans*, mouse, rat, and *Drosophil*a genomes (419, 539, 517, and 340 genes, respectively (Table 2)). The KEGG database contains 383, 333, and 170 lipid metabolic genes for the mouse, rat, and *Drosophila* genomes, respectively. After combining both sets of lipid genes and discarding any overlapped gene in each organism. In total, 585, 563, and 428 lipid genes could be identified in mouse, rat, and *Drosophila* genomes, respectively, with the additional 581 lipid genes within the human genome (Additional file 3: Table S2 and Additional file 4: Figure S2).

### Annotation of lipid metabolic genes in *C*. *elegans*

The 168 lipid metabolic genes we retrieved from the KEGG database were already annotated. The remaining 303 lipid genes were annotated via Ensembl BioMart (http://asia.ensembl.org/biomart/martview/53ac7015dbd78f9a5c2f0a3479fa4dc4),  Swiss-Prot/TrEMBL (http://www.expasy.org), and Gene Ontology (http://www.geneontology.org/). However, since the annotation databases are incomplete, if a gene could not be annotated from a database, they were annotated by hand using previously published papers (primarily retrieved from PubMed) and WormBase (http://www.wormbase.org/). Following annotation, the biological functions of all 471 genes were manually back checked via WormBase.

The biological processes, KEGG path, gene cellular localization, and molecular function annotation of the 471 genes were processed through ClueGo [29] plug-in of Cytoscape [87].

### Retrieval and comparison of disease associated genes in humans, mice, rats, *C*. *elegans*, and *Drosophila*

From the NCBI database (ftp://ftp.ncbi.nih.gov/gene/DATA/), 19994 MIM disease ID genes, phenotypes, and locus were downloaded from NCBI ftp. In total, 15181 genes could be mapped by Ensembl Biomart ID Mapping and were therefore considered human disease genes. All metabolic disease genes were searched from OMIM database (http://www.ncbi.nlm.nih.gov/omim/) using the following keywords: Obesity, Diabetes, Metabolic disease, Sleep apnea, Atherosclerosis, Coronary disease, Hyperlipidemia, and Hypertension. After excluding redundant genes, some further 1264 human metabolic disease genes were identified in our database.

Orthologs information of human gene inferred by Ensembl (release 66) ortholog detection pipeline was obtained from Ensemble’s BioMart interface (http://asia.ensembl.org/biomart/martview/53ac7015dbd78f9a5c2f0a3479fa4dc4). Specifically, we retrieved Ensembl identifiers of orthologs to human genes together with identifiers of their translated protein products. Details of the ortholog detection pipeline are described at http://aug2007.archive.ensembl.org/info/data/compara/homology_method.html. Briefly, gene families were identified from all sequences in the database by WU-Blastp and Smith–Waterman searches, followed by construction of a phylogenetic tree for each gene family to identify orthologous and paralogous relationships between gene pairs.

All orthologs of lipid metabolism genes, human disease genes, and human metabolic genes were then compared by using VENNY, an interactive tool for comparing lists with Venn diagrams (http://bioinfogp.cnb.csic.es/tools/venny/index.html).

### Worm strains, growth condition, and RNA interference

Standard protocols were used to maintain the *C*. *elegans* N2 strain growing at 20°C with *E*. *coli* OP50 for food, unless specifically noted. RNAi was performed by feeding bacterial strains from the Ahringer *C*. *elegans* RNAi library, obtained from Gene Services (Source BioScience) [88,89]. The empty vector L4440 in the Ahringer library *E*. *coli* HT115 strain was used as the negative control for RNAi experiments. The current Ahringer *C*. *elegans* RNAi library covers about 86% of both known and predicted genes. In our *C*. *elegans* lipid metabolism genes database, only 356 genes have available RNAi bacterial strains. Generally, eggs were isolated from gravid adults using hypochlorite treatment and hatched in M9 buffer overnight, and then synchronized L1 worms plated onto NGM plates seeded with *E*. *coli* strain HT115 carrying the RNAi clone of a specific lipid gene. Worms were occasionally checked after 48 hr. RNAi of 356 genes was carried out for four biological rounds.

### Nile Red staining of fixed nematodes to visualize fat storage

Late L4s or young adult nematodes with one or two eggs within their uterus were washed off growth plates, fixed and then stained with Nile Red as described previously [48,90]. All images were captured using identical settings and exposure time.

### Growth rate and sterility analysis

Growth rate analysis was conducted in line with our previous report [90]. All images were captured using identical settings and exposure times. Worms which clearly laid fewer eggs were recorded as having a “egg laying defect”, and those with no eggs were recorded as “sterile”.

## Competing interests

All authors declare no conflict of interest.

## Authors’ contributions

Conceived and designed the experiments: BL, YR Z. Performed the experiments: YR Z, YH D, XJ Z, HZ W, XY W. Analyzed the data: BL and YR Z. Contributed reagents/materials/analysis tools: BL, YR Z, YH D, XJ Z, HZ W, XY W. Wrote the paper: BL and YR Z. All authors have read and approved the final manuscript.

## Supplementary Material

Additional file 1: Table S1 *C*. *elegans* lipid metabolic gene database. 471 lipid metabolism genes were retrieved from the *C*. *elegans* genome. Different colors were used to highlight the origin of the various lipid genes. The metabolism pathways of each gene mainly come from the KEGG database. Click here for file

Additional file 2: Figure S1 Annotation of biological processes of 471 *C*. *elegans* lipid metabolism genes using ClueGO. The chart displays part of significant enrichment analysis of Gene Ontology molecular function in *C*. *elegans* lipid metabolic gene database. The x axis stands for the amount molecular function terms in Gene Ontology. One star denotes P < 0.05, while two stars denote P < 0.01. Click here for file

Additional file 3: Table S2 Lipid metabolism genes in 5 model organisms. All genes IDs were unified as Ensembl gene ID. Respectively, 581, 585, 563, and 428 lipid metabolic genes were present in the human, mouse, rat, and *Drosophila* genomes. Click here for file

Additional file 4: Figure S2 The list of human, mouse, rat, and *Drosophila* lipid metabolic genes. 581 (A), 585 (B), 563 (C), and 428 (D) lipid metabolism genes present in human, mouse, rat, and *Drosophila* genome, respectively. Abbreviation: CEL- *C*. *elegans*; DRO- *Drosophila*. Click here for file

Additional file 5: Table S3 Lipid metabolism genes related to human metabolic diseases in humans, *C*. *elegans*, mice, rats, and *Drosophila*. Respectively, 97, 94, 128, 107, and 97 lipid genes were overlapped with metabolic diseases in human, *C*. *elegans*, mouse, rat, and *Drosophila*. Click here for file
