# Supplementary figures and images for: Comparative genomics and functional study of lipid metabolic genes in Caenorhabditis elegans
Source: BMC Genomics. 2013 Mar 12;14:164. doi: 10.1186/1471-2164-14-164 (PMC3602672; doi:10.1186/1471-2164-14-164)

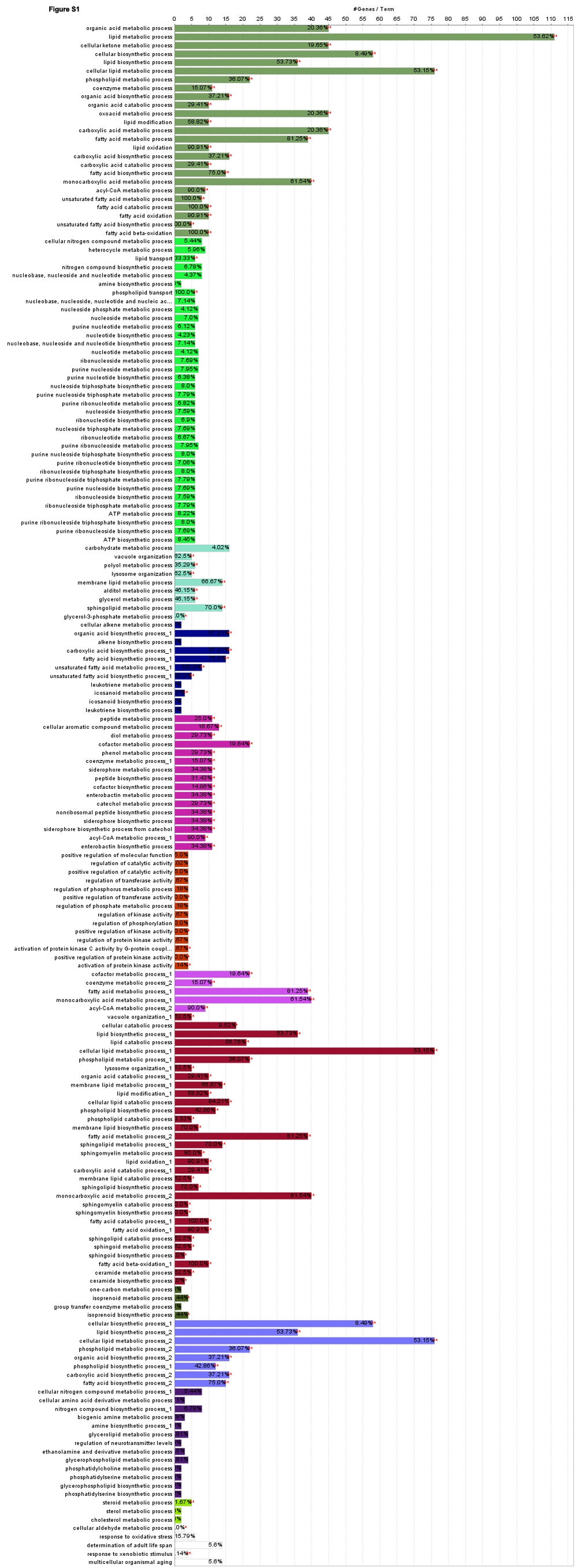

Supplement: Additional file 2: Figure S1 — Annotation of biological processes of 471 C. elegans lipid metabolism genes using ClueGO. The chart displays part of significant enrichment analysis of Gene Ontology molecular function in C. elegans lipid metabolic gene database. The x axis stands for the amount molecular function terms in Gene Ontology. One star denotes P < 0.05, while two stars denote P < 0.01. [file 1471-2164-14-164-S2.tiff]

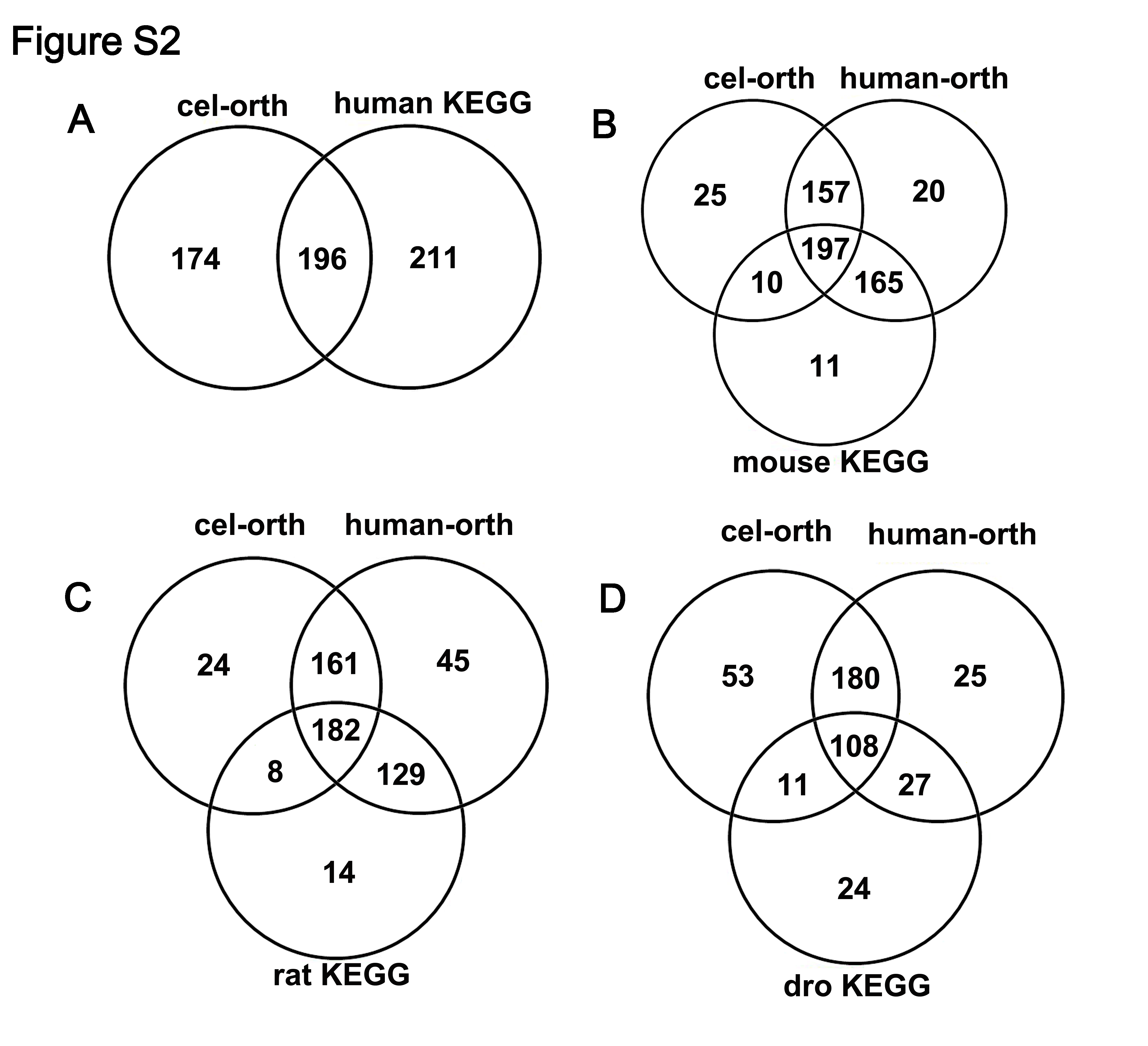

Supplement: Additional file 4: Figure S2 — The list of human, mouse, rat, and Drosophila lipid metabolic genes. 581 (A), 585 (B), 563 (C), and 428 (D) lipid metabolism genes present in human, mouse, rat, and Drosophila genome, respectively. Abbreviation: CEL- C. elegans; DRO- Drosophila. [file 1471-2164-14-164-S4.tiff]
